# Long Term Outcomes in Idiopathic Inflammatory Myositis: An Observational Epidemiologic Study over 15 Years

**DOI:** 10.31138/mjr.280823.lto

**Published:** 2023-08-28

**Authors:** Ramya Janardana, Sangeetha KN, Vasudha Bhat, Divya Balakrishnan, John Michael Raj, Benzeeta Pinto, Chanakya K, Raghunandan Nadig, Anita Mahadevan, Vineeta Shobha

**Affiliations:** 1 Department of Clinical Immunology and Rheumatology, St. John’s Medical College Hospital, Bengaluru, Karnataka, India,; 2 Department of Biostatistics, St. John’s Medical College Hospital, Bengaluru, Karnataka, India,; 3 Department of Neurology, St. John’s Medical College Hospital, Bengaluru, Karnataka, India,; 4 Department of Pathology, NIMHANS, Bengaluru, Karnataka, India

**Keywords:** idiopathic inflammatory myositis, mortality, functional outcomes, clinical response, steroid withdrawal

## Abstract

**Background:**

We report a longitudinal observational cohort of idiopathic inflammatory myositis (IIM) focusing on the long-term clinical outcome and associated parameters.

**Methods:**

IIM patients were classified as per Bohan and Peter criteria. In those with ≥ 24 months of follow-up; the treatment response, functional outcomes, and damage at last follow-up were recorded. Complete clinical response and clinical remission as defined by Oddis et al., was used to define outcomes at last follow-up.

**Results:**

The cohort consists of 175 patients, mean age 40.9 (+12.6) years, M:F 1:3.3; and the major subsets were dermatomyositis (44.6%), overlap myositis (25.7%), antisynthetase syndrome (6.3%), polymyositis (14.3%), and juvenile DM/OM (8.6%). Ninety-four patients have followed up for 24 months or more, with the median (IQR) of 65(35,100.7) months. Of them, 74.1% and 11.8% had complete and partial clinical responses respectively at the last follow-up. In our cohort 40.2% were off-steroids and 13.8% were in clinical remission at the last follow-up. Complete clinical response was associated with better functional outcomes and lesser damage as determined by HAQ-DI of 0[OR10.9; 95%CI (3.3,160)], MRS [OR 3.2; 95%CI (1.4,7.3)] and lesser MDI [OR 1.7; 95% CI (1.1,2.7)] respectively as compared to partial response (unadjusted analysis). Baseline parameters and IIM subsets did not significantly influence the functional outcome and damage. The mortality rate in our cohort is 24/175 (13.7%), the disease-specific mortality rate being 9.1%. Large majority of deaths were early, associated with active disease.

**Conclusion:**

We report good long-term outcomes in all major myositis subsets. Partial clinical response to treatment is associated with worse functional outcomes and damage accrual. Death occurs early in association with active disease.

## KEY MESSAGES

1. At 24 months follow-up, the outcome is favourable with immunomodulatory treatment and glucocorticoids can be discontinued in 40.2% of IIM patients.
2. Autoantibodies and the clinicopathological subsets did not influence the outcome in our cohort.
3. Mortality is early and is disease-related.

## BACKGROUND

Idiopathic Inflammatory myositis (IIM) is a group of potentially treatable, rare heterogenous chronic systemic autoimmune disorders predominantly affecting the skeletal muscles. A multitude of immunological perturbations contributes to the pathogenesis of myositis, leading to varying clinical phenotype and severity of not only the myositis, but also that of extramuscular organs chiefly lung and skin.^1^ The classifications of IIM, definitions of its clinical phenotypes, autoantibody profiling, and reporting pattern for muscle biopsy have undergone an immense overhaul in last few decades as our understanding of contributory mechanisms has expanded. In parallel, the myositis core set measures have also been updated for outcome as well as for response; however, they remain time-consuming and difficult to employ in clinical as well as research settings.^2,3^

Description of large cohort studies with long-term outcome and predictors for response can support and direct research to improve therapeutic results.^4,5,6^ Prospective multicentric cohorts have been initiated at various centres in Europe and the US, and outcome data from them should be available in near future.^5,7^ Further, there is an influence of distinct geographic, ethnic, and/or environmental factors on the clinical phenotype and the autoantibody distribution, underpinning the need for long-term outcome studies across the world.^8^ From the Indian subcontinent, the IIM cohorts with long-term follow-up are rather sparse.^9-14^ Here, we describe the long-term outcome, prognostic factors, and mortality data of the IIM subsets over 15 years from our tertiary care referral medical college hospital, India using standard measures of clinical response and patient-reported outcomes.

## METHODS

### Study design

This is a longitudinal cohort study carried out from 2006 to 2020 in a tertiary care referral centre. A structured case report form (CRF) has been employed to record the clinical phenotype, immunosuppressive treatment, and outcomes on follow-ups for all consecutive patients of IIM in real-time. All patients were classified as definite/probable/possible IIM as per Bohan & Peter criteria.^15,16^ Since data has been recorded over the last 15 years, the new ACR/EULAR classification criteria have not been applied to classify patients; however, this has been studied in the same cohort by our group previously and published.^13,17^

## CASE SELECTION

All the CRFs were scrutinised and patients were categorised into dermatomyositis (DM), polymyositis (PM), overlap myositis (OM), antisynthetase syndrome (ASS), immune-mediated necrotising myositis (IMNM), juvenile-onset myositis, and clinically amyopathic DM (CADM: includes both amyopathic and hypomyopathic subsets).^17-21^ Association with malignancy was defined by diagnosis of malignancy within 3 years of onset of myositis. Other causes of myopathy such as drug-induced, metabolic myopathy, muscle dystrophy, and inclusion body myositis were excluded. The pharmacotherapy included glucocorticoids and upfront steroid-sparing immunosuppressants (IS) in all patients as per the clinician’s judgment. The study was approved by the Institute Ethics Review Board (IERB 80/2017).

### Disease specific evaluation and laboratory assessments

The initial assessments consisted of various descriptors of clinical phenotype including manual muscle testing (MMT-8), myositis intention to treat activity index (MITAX), and dermatologist opinion of cutaneous manifestations.^2,22^ Extramuscular organ assessment was as per clinical indication. Laboratory assessments include measures of muscle inflammation such as creatine kinase enzyme (CK) level, aspartate aminotransferase (AST), and lactate dehydrogenase (LDH). Autoantibody profiling has evolved and expanded sequentially with time, consisting of antinuclear antibody (ANA) by indirect immunofluorescence (IIF), myositis associated antibodies (anti RNP/Sm, anti Ro-60, anti La, anti Ro-52) and anti Jo-1 (myositis specific antibody) as part of EUROIMMUN ANA profile-3 immunoblot assay. In a subset of cohort, EUROIMMUN myoblot was performed which includes anti Mi2, anti SRP, anti aminoacyl t-RNA synthetase antibodies (ARS), anti Pm-Scl and anti Ku. All immunoblot strips were analysed with the EUROLineScan (Euroimmun) and +, ++ or +++ was considered significant. EMG was performed by a neuro physician at our centre. Muscle biopsy was performed in consenting patients and were analysed by neuropathologist at NIMHANS (National Institute of Mental Health and Neurosciences) using haematoxylin and eosin stain, modified Gömöri trichrome stain, periodic acid Schiff (PAS), and immunohistochemistry, as considered appropriate.

### Outcome assessment

We report outcomes in those who had been followed for at least 24 months. Assessments at baseline and each follow-up visit included evaluation of muscle power, the status of extra muscular features and laboratory parameters. These are categorised as complete clinical response, clinical remission, and partial clinical response as per Oddis et al.^23^ Complete clinical response was defined as lack of evidence of active myositis while still receiving therapy for ≥6 months. Clinical remission was defined as lack of evidence of myositis while not receiving any drug therapy for ≥6 months.^23^ Patient outcomes were classified as partial clinical response if they did not satisfy the above definitions of complete clinical response/clinical remission. Relapse was defined as clinical/laboratory worsening after a period of improvement requiring treatment alterations.^24^

Functional disability at the last follow-up was assessed using MD-health assessment questionnaire (MD-HAQ) a patient-reported measure and modified Rankin score (MRS), patient disability as assessed by the trained clinician or nurse.^25,26^ Damage accrual at last follow-up was assessed using Myositis damage index (MDI).^2^ Mortality was classified as ‘myositis related’ based on disease activity in any of the domains.

### Statistics

Demographic details were represented as frequency (percentage) mean (+/- standard deviation) and median (interquartile range) as appropriate. The Chi-square test or Fisher exact test was used to check for an association between categorical variables. Association studies for means of continuous variables were evaluated using Student’s t-test or Wilcoxon rank sum test as appropriate. Factors associated with complete clinical response, steroid withdrawal, and mortality were analysed using logistic regression analysis. Factors found to be significant (p<0.05) in univariate analysis were taken up for multivariate analysis. Survival function was analysed using Kaplan Meier estimation. STATA software version 16 was used for the above analysis, p-value was kept at 5% significance.

## RESULTS

We reviewed a total of 190 CRFs, 15 were excluded from current analysis due to incomplete information or change in diagnosis at follow-up. The mean age of the cohort (n=175) was 40.9 years (+12.6) with female preponderance (F:M::3.3:1). Majority [133/175(76%)] were definite/probable IIM as per Peter and Bohan criteria and the rest were classified as possible IIM which largely included overlap myositis and CADM. The baseline demographics, disease subsets, muscle enzymes, autoantibody profile, and muscle histopathology features are represented in **Supplementary Table 1**.

**Table 1.** Outcome parameters at ≥ 24 months of follow-up in IIM subsets (n=94; Median follow up duration 65 months).

	**Overall (94)**	**DM (35)**	**OM (33)**	**PM (13)**	**JM (8)**	**ASS (5)**
	n (%)	%	%	%	%	%

**Course**						
Monocyclic	13(13.8)	10.8	15.2	23.2	12.5	0
Polycyclic	35(37.2)	43.3	33.3	30.7	50	40
Chronic continuous	46(48.9)	45.9	51.5	46.1	37.5	60

**Response**						
Complete clinical response	69(74.1)	80.6	75.7	53.8	62.5	100
Clinical remission	13(13.8)	10.8	15.2	23.1	12.5	0
Partial clinical response	11(11.8)	8.6	9.1	23.1	25	0

**GC dose** (mg)/day **at last FU** (n=87)						
0	35(40.2)	42.4	43.3	38.5	42.8	0
≤7.5	45(51.7)	51.5	46.7	53.8	42.8	0
>7.5	7(8.1)	6.1	10	7.7	14.4	100

**HAQ(n=73)**						
**0**	45(61.6)	65.5	64.3	45.5	NA	60
**1**	24(32.8)	34.5	32.1	36.4		20
**>1**	4(5.4)	0	3.6	18.1		20

**MDI(n=90)**						
**0**	38(42.2)	48.6	34.4	36.4	50	50
**1-2**	36(40.1)	37.1	40.6	54.5	25	50
**>2**	16(17.7)	14.3	25	9.1	25	0

**MRS (n= 84)**						
**0-1**	61(72.6)	72	65.5	58.3	100	80
**2**	19(22.6)	28	31	25	0	0
**>2**	4(4.8)	0	3.5	16.7	0	20

HAQ: Health Assessment questionnaire; MDI: Myositis Damage Index; MRS: Modified Rankin Score; FU: Follow up; Rx: Treatment; GC: Glucocorticoid, NA: Not applicable.

**Figure 1.**
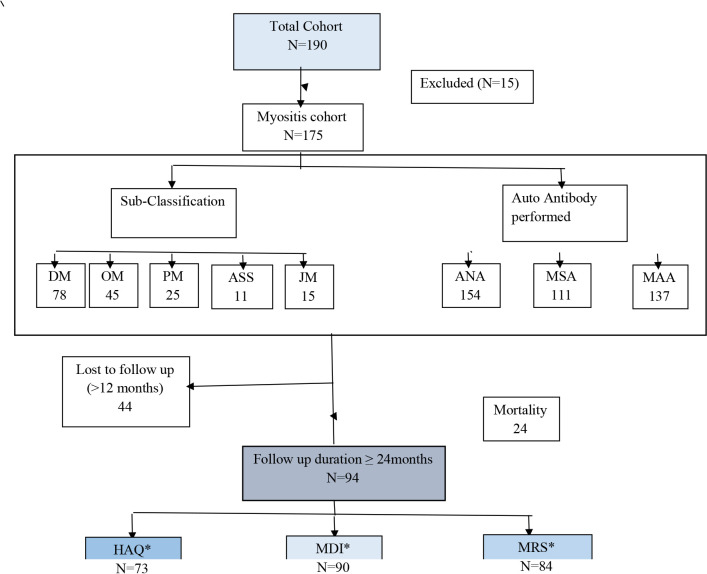
Schematic representation of myositis cohort. ANA: Antinuclear Antibody; ASS: Antisynthetase Syndrome; DM: Dermatomyositis; HAQ: Health Assessment Questionnaire; JM: Juvenile Myositis; MAA: Myositis Associated antibody; MDI: Myositis Damage Index; MRS: Muscle Ranking Score; MSA: Myositis Specific Antibody; PM: Polymyositis; OM: Overlap Myositis. *Refers to data available at last follow up.

They were sub-classified as DM 78(44.6%), OM 45 (25.7%), ASS 11(6.3%), and PM 25(14.3%), and a single case of necrotising autoimmune myopathy. In the subset with DM, 5 were CADM. Juvenile onset IIM was categorised into juvenile DM (12/15) and juvenile-onset OM (3/15). Association with malignancy was noted in 7 patients (DM-5, CADM-1, PM-1). The malignancies noted were carcinoma of breast (2), papillary carcinoma of thyroid (1), lung carcinoma (2), and adenocarcinoma of ovary (2). Overall schema of our cohort is represented in **Figure 1**.

### Myositis related autoantibodies

ANA was detected in 97/154 (63%). MSAs were demonstrated in 26/111(23.4%), most frequent being Jo-1 12/111 (10.8%), Mi-2 10/111 (9%) and non-Jo-1 ARS 8/111 (7.2%). MAAs were detected in 81/137 (59.1%) while 16 (11.7%) were negative for both MSA & MAA. Multiple MSA positivity was seen in 6/111 (5.4%), an overlap of MSA and MAA was noted in 11/111 (9.9%). These have been detailed in **Supplementary Table 1**.

### Clinical course and factors associated with steroid discontinuation

All patients were treated with steroids. The distribution of steroid sparing IS is represented in **Supplementary Table 1**. Methotrexate was the most common steroid sparing agent prescribed in 90 (52%) of patients, the median number of steroid sparing IS being 1(1,2). Nineteen (10.9%) patients were administered rituximab due to either relapse or inadequate response.

We recorded the follow-up information at >24 months in 94(54.9%) patients. At the last follow-up [65(35,100.7) months], 69(74.1%) were in complete clinical response, clinical remission was attained in 13(13.8%) and 11(11.8%) had a partial clinical response.

### Clinical response

Complete clinical response/clinical remission at the last follow-up was achieved by 82/94 (87.2%) patients. They were less likely to have had a relapse [OR 4.9(1.2,19.8)]. This group had HAQ-DI of 0 [OR 10.9(3.3,160)], better MRS [3.2(1.4,7.3)] and lesser MDI scores [1.7(1.1,2.7)] as compared to those who had partial response [11(11.8%)] (**Table 2**).

**Table 2.** Prognostic factors for clinical response, steroids discontinuation, and mortality.

	**Odds ratio (95% CI)**	**P-value**	**Adjusted odds(95% CI)**	**P-value**
**Complete clinical response and/or clinical remission at last follow-up (n=93)**				
**No Relapses**	4.9(1.2, 19.8)	0.02	12.9(0.9,188)	0.06
**HAQ ≤1 at last f/u**	10.9 (3.3, 160)	<0.01	6.7(0.8,51.8)	0.06
**MRS**	3.2(1.4,7.3)	<0.01	1.7(0.4,6.6)	0.4
**MDI**	1.7(1.1,2.7)	0.01	1.7(0.9,2.9)	0.07
**Steroid withdrawal (n=93)**				
**Myositis associated antibody**	5.7(1.**8**,17.4 )	<0.01	4.6(0.69,30.6)	0.14
**Ro 52**	3.2(1.1,9.5)	0.03	1(0.14,6.9)	0.9
**Follow-up duration**	0.9(0.96,0.99)	<0.01	0.9(0.96,0.99)	0.02
**HAQ <1 at last f/u**	3.3(1.3,8.6)	0.01	1.8(0.2,14.7)	0.5
**MRS at last f/u**	1.7(1,3)	0.03	1.2(0.3,4.7)	0.7
**Mortality (n=175)**				
**DM vs non-DM**	2.52(0.9,6.3)	<0.01		
**Baseline MMT-8**	0.97(0.93,1)	0.05	0.9(0.9,1.1)	0.1
**Dysphagia**	2.5(1.03,6.2)	0.04	1.5(0.3,6.8)	0.6
**Dysphonia**	5.3(1.6,17)	<0.01	3.4(0.3,35.8)	0.3
**Respiratory weakness**	6.3(1.8,22)	<0.01	2.5(0.2,28.3)	0.4
**Malignancy**	6(1.9,18.3)	<0.01	1.5(0.2,10.5)	0.7
**RNP/Sm positivity**	0.12(0.02,0.9)	<0.01	0.1(0.02,1.2)	0.07

Factors analysed in univariate analysis were age, sex, diagnosis, time to presentation, baseline MMT-8, oropharyngeal weakness, ILD, MSA positivity, MAA positivity, Ro-52 positivity, RNP/Sm positivity, time to 0-15 mg/kg steroids, response at 6 months, relapsing disease, HAQ-DI, MRS, MDI at last follow-up. Significant factors (p=<0.05) only are represented in the table. DM: Dermatomycosis; MMT8: Manual Muscle Testing; MRS: Muscle Ranking Score; MITAX: myositis intention to treat activity index; HCQ: Hydroxychloroquine, CR: Complete Response.

Clinical remission was attained by 13 (13.8%) individuals, after a median (IQR) of 60 (36,89) months. They were less likely to have dysphagia [OR 0.3(0.09,0.9)], were Ro-52 positive [OR 8.5(1,70)], had moderate severity at presentation [median (IQR) MMT-8 score-52.5 (50,59)].

Overall, complete discontinuation of steroids could be achieved in 35 (40.2%) and another 45 (51.7%) were on low dose prednisolone <7.5mg/day (**Table 1**). Complete discontinuation of steroids was associated with MAA positivity ([OR 5.7(1.8,17.4)] specifically, Ro-52 positivity [OR 3.2(1.1,9.5)], better HAQ-DI [OR 3.3(1.3,8.6)] and MRS [OR 1.7(1,3)]) scores and a longer follow-up duration [OR 0.9(0.96,0.99)] as compared to those who were on some dose of steroids (**Table 2**). Except for a longer duration of follow-up, none of the other factors had an independent association with steroid discontinuation.

**Figure 2.**
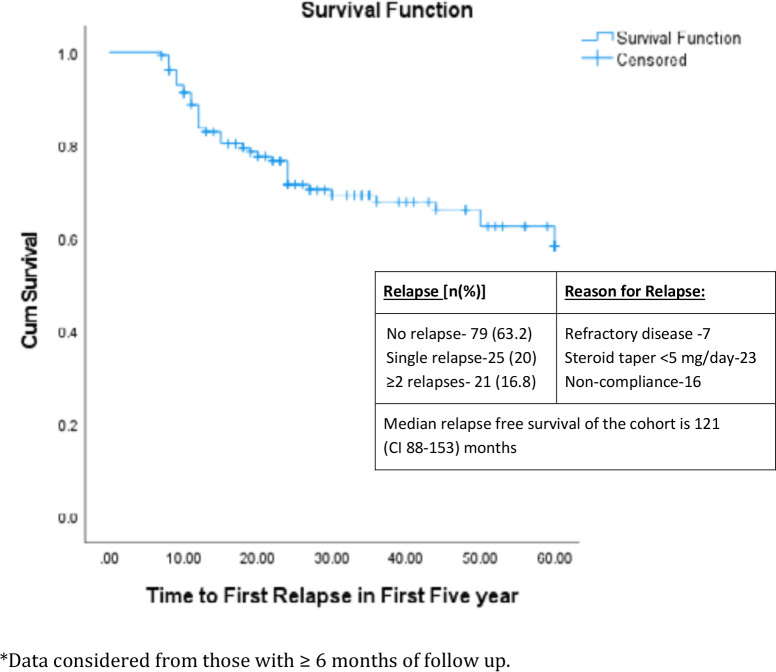
Relapse-free survival curve (n=125)*.

The median relapse-free survival rate of our cohort is 121 (CI 88-153) months (**Figure 2**). Factors such as IIM subtype, dysphagia, ILD, MAA, complete vs partial response, median HAQ, median MRS, and median MDI were not different in the subgroups who had at least one relapse versus those who had no relapse.

### Functional outcome

**Figure 3.**
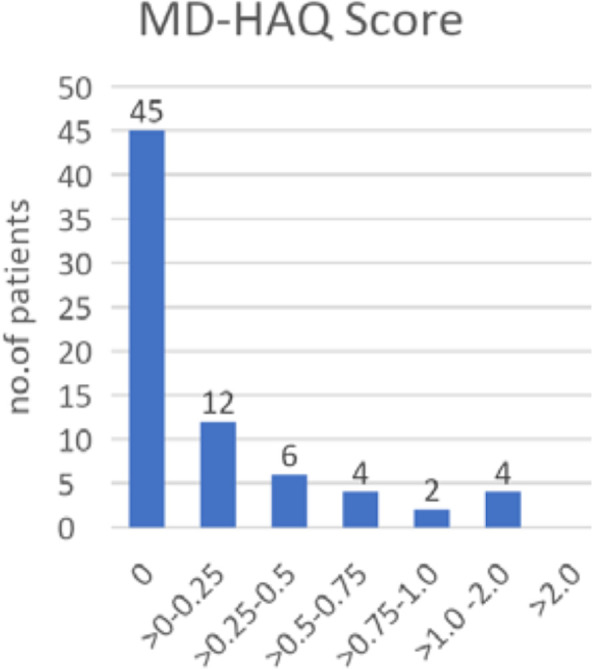
MD-HAQ score at last follow-up (median duration of follow-up 65[35,100.7] months).

Median HAQ score recorded at last follow-up was 0(0,0.2). There was no disability in 45 (61.6 %), another 24(32.8 %) patients had mild to moderate disability (HAQ >0 <1). (**Table 1**, **Figure 3**). Correspondingly, MRS scores were favourable in our cohort with median scores of 0(0,2); 61(72.6%) having no residual symptoms or no significant disability(score-0,1). The median MDI score of the cohort was 1(1,2).

### Mortality

**Figure 4.**
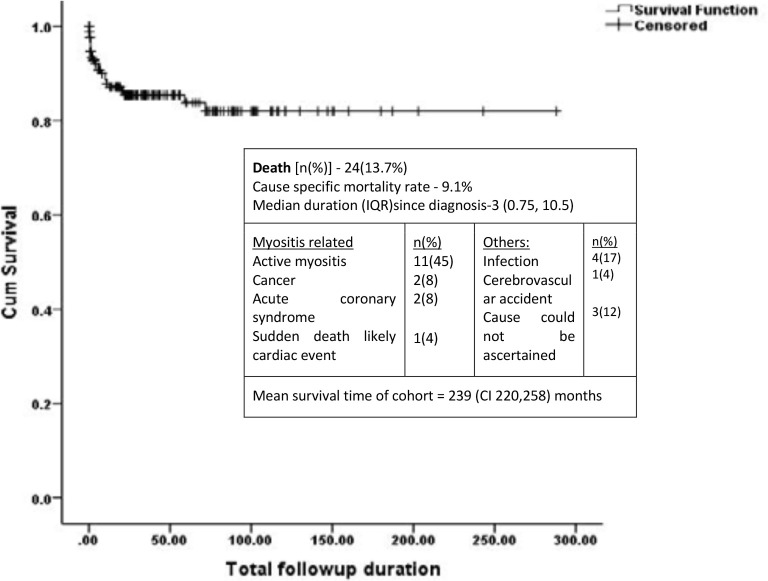
Cumulative survival curve of the cohort.

Overall, 24/175 (13.7%) patients have died, and the disease-specific mortality rate was 9.1%. One year, 5 years and 10 years cumulative survival rate of our cohort was 89.1%, 86.9%, and 86.3% respectively and the mean survival time was 239 (CI 220,258) months. (**Figure 4)**. Majority of the deaths (15/24) occurred during the initial 6 months of illness and were related to disease (45.8%) or infectious complications (16.6%). The median duration from diagnosis to death was 3 (0.75,10.5) months. Univariate regression analysis of factors associated with mortality were baseline MMT-8 [OR 0.97(0.93,1)], dysphonia [5.3(1.6,17)], dysphagia [2.5(1,6.2)], respiratory weakness [6.3(1.8,22)], malignancy [6(1.9,18.3)], and presence of RNP/Sm [0.1(0.02,0.9)]; however, none of the factors were found to be significant in multivariate analysis (**Table 2**).

## DISCUSSION

We present single-centre longitudinal data of inflammatory myositis from the Indian subcontinent in a real-life setting. The clinical phenotype of our cohort is similar to the published reports across the world; however, we report a lower prevalence of extra muscular manifestations (**Supplementary Table 2**).^4,5,10,27^ The higher female preponderance and lesser mean age in our cohort as compared to EuroMyositis cohort is likely contributed by a higher prevalence of OM subset, however, it is comparable to the other Indian and Chinese cohorts.^4,5,10,27^ Polymyositis proportion (14.3%) is higher than in other published cohorts, probably attributable to a prevalent understanding of IIM a decade before. We have learned since the discovery of MSA and recognition of MAA, that more and more PM can be classified either as DM or ASS or IMNM or IBM.^28^

The induction and maintenance of immunosuppressive treatment is in line with other published cohorts. Early institution of steroids-sparing immunosuppressive agents was employed in all patients. The hallmark of our cohort is the discontinuation of steroids in almost one-third of patients after a median of 65 (35,100.7) months and the ability to reduce it to <7.5 mg/day prednisone in close to half (47.9%) of the patients. There are very few studies discussing this matter, however, it is comparable to a large Chinese cohort.^4^ Complete clinical response and clinical remission were associated with an absence of relapse, better functional outcomes (HAQ-DI and MRS), and reduced damage accrual in our cohort. We believe that diligent matching of disease status and aggressiveness of IS therapy over a long period of time may be the key determinant.

Clinical remission differs in various cohorts. Clinical remission was around 20% in the Netherland cohort and was not associated with any of the factors studied.^26^ Another Indian Cohort by Ramesha et al. has described clinical remission in 39/68 (57%) of their cohort, albeit in a smaller cohort size with a lesser duration of follow-up.^29^ Clinical remission in our cohort was 13.8%; it was associated with the absence of dysphagia at diagnosis, Ro52 positivity and a longer duration of follow-up. Clinical remission is a subset, which needs dedicated research in future prospective cohorts.

Significant disability at the last follow-up is seen in only <5% of patients in our cohort, which is notably different from the Netherland (24%) and Hungarian cohorts (47%).^26,30^ Furthermore, in comparison to the Chinese cohort as well, the median HAQ-DI score in our cohort is lesser even though the number of patients with a mild disability is higher, thereby demonstrating an overall good outcome. Both the Caucasian cohorts were published a decade and half earlier, the improved outcomes in ours and the Chinese cohort may be reflective of early referral, and a better understanding in the management over the last 2 decades. Amongst those who have survived, unless there is a recent relapse (<10%), patients have remained quiescent on or off medications, which is similar to the Chinese and Hungarian cohort.^4,31^

Disease-specific mortality rate (9.1%) in our cohort is comparable to other large cohorts.^4,5^ Mortality rate and aetiologies of death differs in various cohorts depending on the definition of cause of death, inpatient vs population-based mortality data. The majority of deaths (62.5%) occurred early (<6 months since diagnosis); due to active disease and/or infections. A similar observation has been noted in recent publications from India too.^9,11^

Furthermore, infections as a major cause of mortality has also been highlighted in most large cohorts viz. Chinese, US, Mexico, Japanese, and Spanish (20-60%) cohorts too.^31-34^ However, in some other cohorts (Hungarian, Swedish and Netherland cohorts), active disease leading to cardiorespiratory involvement and malignancy forms the leading cause of death.^33,34^ Higher infections in our IIM cohort may be related to the intense initial immunosuppression and the general living conditions of our population. Although factors such as diagnosis of DM, oropharyngeal weakness, MMT-8, respiratory weakness, and malignancy were found in association with mortality in univariate analysis in our cohort, none of these factors remained significant in multivariate analysis. Age at onset, malignancy, cardiopulmonary and respiratory muscle involvement appeared as risk factors to death in a few cohorts.^4,26,35^ Overall, contributing factors for mortality in IIM deserve attention on a larger scale, to ascertain and ameliorate modifiable factors. With easier availability of extended autoantibody profiling in IIM, this association may prove to be the most determining factor.

## LIMITATIONS

Over the last few decades, understanding of the interrelationship between clinical phenotypes, autoantibody associations, immunopathogenesis, and therapeutic options for IIM have undergone a substantial transformation. The autoantibody assays, methodologies and the therapeutic choices have been variable as per prevalent opinions and consensus in this real-life experience cohort. Some of the IIM subsets were small to accurately examine the impact on outcome. In the subgroup with relapse, a structured review of compliance has not been performed. Similarly, in the mortality subgroup, information about terminal events and their treatment is unclear in some.

## CONCLUSIONS

IIMs are potentially treatable diseases, requiring long-term treatment and follow-up. Through our study, we emphasise that good long-term functional outcomes as determined by low HAQ, MRS can be achieved in majority. Complete clinical response and discontinuation of steroids is associated with better functional outcomes. Damage accrual is more in those who are unable to achieve complete clinical response with treatment. Infection remains an important cause of early mortality second only to active disease.

## DECLARATION

This article was published in research square pre-print (without a peer review process) with a DOI 10.21203/rs.3.rs-598502/v1; however, it is currently not under peer review process in any other journal.

## IEC APPROVAL

Study is approved by Institutional Ethics Committee St. John’s Medical College, St. John’s National Academy of Medical Sciences.

## Figures and Tables

**Supplementary Table 1.** Overview and distribution of IIM subsets, clinical phenotype, laboratory parameter, autoantibodies, treatment, and mortality.

	**Overall**	**DM**	**OM**	**PM**	**JM**	**ASS**

**N (%)**	175	78(44.6)	45(25.7) 20/11/10/4 (SSc, MCTD, UCTD, SLE)	25(14.3)	15(8.6)	11(6.3)

**Mean age in Years (SD)**	40.9 (+12.6)	40.5(+13.4)	39.1(+11.4)	41.9(+13.4)	12.3(+3.6)	47.9(+9.6)

**F:M**	3.3:1	2.9: 1	6.5:1	3.2:1	2:1	1.2:1

**Median (IQR) duration of illness(months)**	4(1,7)	3(1,8)	4(1,5)	4(1,6)	2(1,6)	3(2,14.5)

**Median MMT-8 (baseline) n=133**	61(49,69)	61(50,67)	61(49,72)	55(39,61)	61.5(45.5,71)	66(61.5,75)

**MITAX (baseline) n=75**	12(7,16)	12.5(7,18)	12(6.75,12)	9(5,12)	13(8.5,15.5)	0

**Muscle enzymes**						
Mean (SD) Creatine	3975(7206)	3103(5259)###	3250(2753)	8827(13912)	2818(8244)	3950(5766)
Kinase (U/L)						
Mean (SD)Aspartate aminotransferase(U/L)	206(412)	168(196)	141(104)	435(931)	283(471)	116(108)*

**Dysphagia (%)**	50 (28.6)	27(34.6)	14 (31.1)	6 (24)	3 (20)	0 *

**Dysphonia (%)**	13 (7.4)	5 (6.4)	4 (8.8)	2 (8)	2 (13.3)	0

**Respiratory weakness(%)**	6 (3.4)	2(2.5)	4 (8.8)	0	0	0

**ILD (%)**	24 (13.7)	6 (7.7)	10 (22.2)*	1 (2.4)	1 (6.6)	6(54.5)***

**DM specific rash(%)**	70 (40)	54 (69.2)	3 (6.6)	1 (2.4)	10 (66.6)	2(18.2)

**Cardiac (%)**	11 (6.3)	1 (1.3)	6 (13.3)*	0	1(6.6)	3(27.3)**

**Calcinosis (%)**	13 (7.4)	5 (6.4)	2 (4.4)	1(2.4)	3 (20)	2(18.2)

**Malignancy (%)**	7 (4)	6 (7.7)	0	1(2.4)	0	0

**ANA (n=154)(%)**	97 (63)	36(46.1)	34 (87)	10(41.6)	9 (60)	8 (80)

**MSA (n=111)(%)**						
**Jo-1**	12 (10.8)	2(4.4)	1(4.3)	1(6.6)	-	8 (72.7)
**Non Jo-1 ARS**	8 (7.2)	4 (8.8)	-	1 (6.6)	-	3 (27.2)
**MI-2(αβ)**	10 (9)	8 (17)	1 (4.3)	-	1 (7.6)	-
**SRP**	3 (2.7)	1 (2.2)	2 (8.6)	-	-	-

**MAA (n=137) (%)**						
**U1RNP**	41 (29.9)	9 (16.3)	25 (62.5)^**^	5 (26.3)	2 (15.4)	0
**Ro-52**	50 (36.5)	14(25.4)	22 (55)^#^	4 (21)	3 (23)	7 (63)^**^
**Ku**	7 (6.3)	3 (5.4)	3 (7.5)	1 (5.2)	-	-
**Pm-Scl**	10 (9)	4 (7.2)	2 (5)	1 (5.2)	3 (23)	^-^
**Scl 70**	5 (3.6)	1 (1.8)	4 (10)	-	-	^-^
**SSA**	9 (6.5)	2 (3.6)	2 (5)	3 (15.7)	2 (15.4)	^-^
**CENP**	4 (2.9)	2 (3.6)	2 (5)	-	-	^-^

**Muscle Histopathology**	n=74	n=34	n=14	n= 14	n=8	n=4

PFA	22	16	2	0	4	0
Endomysial inflammation	3	1	0	1	0	1
Perivascular inflammation	44	21	8	9	4	2
Necrosis	29	14	8	4	2	0

**Treatment N(%)**						
**Steroid (any dose)**	174(99.3)	77(98.7)	45(100)	25(100)	15(100)	11(100)
**MP pulse**	58(33.1)	27(34.6)	13(28.8)	8(32)	5(33.3)	5(45.4)
**Immunosuppressants**						
**Methotrexate**	90 (52)	40(51.2)	26(57.7)	8(32)	10(66.6)	6(54.5)
**Azathioprine**	52 (29.7)	24(30.7)	17(37.7)	8(32)	2(13.3)	1(9)
**Mycophenolate mofetil**	33 (18.9)	13(16.6)	9(20)	5(20)	2(13.3)	4(36.3)
**Cyclophosphamide**	12 (6.8)	5(6.4)	4(8)	1(4)	-	2(18.2)
**Rituximab**	19 (10.9)	8(10.2)	5(11)	-	1(6.6)	5(45.4)
**HCQ**	28 (16)	13(16.6)	9(20)	-	3(20)	3(27.2)
**Tacrolimus**	7 (4)	2(2.5)	3(6.6)	-	1(6.6)	1(9)
**Leflunomide**	4 (2.2)	1(1.2)	2(4.4)	-	-	1(9)
**IVIg**	4 (2.3)	3(3.8)	-	1(4)	-	-
**PLEX**	2 (1.1)	1(1.2)	-	1(4)	-	-

**Death**	24 (13.7)	15(19.2)	2(4.4)	4(16)	2(13.3)	1(9)

Statistically significant:

* p=.03(ASS vs rest & OM vs rest);

# -.01(OM vs rest);

** p=.02(ASS vs rest & OM vs rest);

*** p=.001(ASS vs rest);

### - p=0.001(DM vs OM).

DM: Dermatomycosis; PM: polymyositis; OM: overlap myositis; SSc: systemic sclerosis; MCTD: Mixed connective tissue disease; UCTD: undifferentiated CTD; SLE: systemic lupus erythematosus; ASS: antisynthetase syndrome; JM: Juvenile Myositis; ILD: Interstitial Lung Disease; MMT8: Manual Muscle Testing; MITAX: myositis intention to treat activity index; PFA: Perifascicular atrophy; HCQ: Hydroxychloroquine; PLEX: Plasma Exchange; IVIg: Intravenous Immunoglobulin; MP: Methylprednisolone.

**Supplementary Table 2.** Comparison with published IIM cohorts from different ethnicities.

Characteristics	Our cohort	Porkodi et al(2002)^22^	Xio Ming Shu et al. (2011)^13^	Euromyositis registry (2017)^36^	KJ chinniah et al. (2020)^42^

n	175	87	188	3067	104

Median(IQR) age at onset	38(27.5,48.5)	25-35	43.8(15.8)	49 (16)	36.8(14.7)

Gender ratio(M:F)	1:3.3	1:2.3	1:2.1	1:2.2	1:4.8

Ethnicity	India	India	China	Caucasian	African and Indian

Median(IQR) disease duration at presentation (months)	4(1,7)	NA	NA	8(3,22)	42(16,103)

Median(IQR) duration of follow up (months)	26.5(6,72)	NA	90	36 (18,72)	33.5(13,100.8)

DM/PM/OM/ASS	78/25/45/11	28/24/30	53/21/14.4/0	31/27/12/17	25/25/40

Juvenile onset	8.6	5	3.7	3	5.8

**Calcinosis(%)**					
Overall JDM	7.4		4.7	6	
	20			44	

Cancer (%)	4	1.1	5.9	13	4.8

MMT-8	61(49-69)	-	-	NA	-

Dysphagia (%)	29.1	33.3	39.9	39	43.4

Dysphonia (%)	8	4.6	11	NA	19.2

Respiratory muscle weakness(%)	3.4	9.2	-	-	-

ILD (%)	13.7	9.2	48.9	30	27

Cardiac (%)	6.3	5.7	34	9	14.4

ANA (%)n=154	63.6	67.3	NA	NA	70.4

Jo-1/Non Jo-1/Mi-2/SRP (N=111)	10.8/7.2/9/2.7	NA	NA	NA	Done in a subset

Ro52/U1RNP/Pm-Scl/Ku (N= 137)	36.5/29.9/9/6.3	NA	NA	NA	Done in a subset<50%

**Treatment (%)**					
Methotrexate	52	57.2		71	50
Azathioprine	29.7	42.8		51	37.7
Mycophenolate	18.9	12.4		23	7.7
Cyclophosphamide	6.8	60		19	10.6
IV Ig	1.1	21.4		12	19.2
Rituximab	16			7	3.8
Median no. of immunosuppressants	1(1,2)			2(1-3)	

**Course (%)** Monocyclic/polycyclic/chronious continuous	13.8/37.2/48.9		15.1/79.5/5.4		

MDI >0(%)	58.2		71.8		
Median	1(1,2)				

**HAQ (%)**					
=0	64.6		83.3		
>0	35.4		16.7		
Median	0(0,0.2)			0.75(0.131.5)	

Death (%)	13.7	11.5	17	NA	7.7

DM: Dermatomycosis; PM: polymyositis; OM: overlap myositis; ASS: antisynthetase syndrome; JM: Juvenile Myositis; ILD: Interstitial Lung Disease; MMT8: Manual Muscle Testing; MITAX: myositis intention to treat activity index; HAQ: Health Assessment questionnaire; MDI: Myositis Damage Index; MRS: Muscle Ranking Score.
